# Targeted and Population-Wide Interventions Are Needed to Address the Persistent Burden of Anemia among Women of Reproductive Age in Tanzania

**DOI:** 10.3390/ijerph19148401

**Published:** 2022-07-09

**Authors:** Bruno F. Sunguya, Yue Ge, Linda B. Mlunde, Rose Mpembeni, Germana H. Leyna, Krishna C. Poudel, Niyati Parekh, Jiayan Huang

**Affiliations:** 1 School of Public Health and Social Sciences, Muhimbili University of Health and Allied Sciences, Dar es Salaam P.O. Box 65001, Tanzania; sunguya@gmail.com (B.F.S.); lindasozy@gmail.com (L.B.M.); rcmpembeni@gmail.com (R.M.); germana.leyna@tfnc.go.tz (G.H.L.); 2 School of Public Health, Global Health Institute, Fudan University, Shanghai 200433, China; 20111020050@fudan.edu.cn; 3 Implementation Science Tanzania, Dar es Salaam P.O. Box 65216, Tanzania; 4 Tanzania Food and Nutrition Center, Dar es Salaam P.O. Box 977, Tanzania; 5 Department of Health Promotion and Policy, School of Public Health and Health Sciences, University of Massachusetts Amherst, Amherst, MA 01003, USA; krishna@schoolph.umass.edu; 6 Institute for Global Health, University of Massachusetts Amherst, Amherst, MA 01003, USA; 7 Public Health Nutrition Program, School of Global Public Health, New York University, New York, NY 10012, USA; np31@nyu.edu; 8 Department of Population Health, NYU Grossman School of Medicine, New York University, New York, NY 10016, USA

**Keywords:** anemia, women of reproductive age, undernutrition, Tanzania

## Abstract

Recent evidence suggests that 44.8% of women of reproductive age (WRA) in Tanzania suffer from anemia. Addressing this public health challenge calls for local evidence of its burden and determinants thereof for policy and tailored interventions. This secondary data analysis used Tanzania Demographic and Health Surveys (TDHS) 2004–2005 and 2015–2016 with a total of 23,203 WRA. Data were analyzed using descriptive statistics to characterize the burden of anemia, regression analyses to examine the adjusted change in the prevalence of anemia and remaining determinants thereof, and the Global Information System (GIS) to map the differences in the burden of anemia in Tanzania over the period of one decade. Considering the risk factors of anemia observed in our study, WRA in Tanzania should have been 15% less likely to suffer from anemia in 2015 compared to 2005. However, a small decline (3.6%) was not evenly distributed across the regions in Tanzania. Factors that remained significantly associated with anemia among WRA in the latest survey include age above 35 years (AOR = 1.564, *p* = 0.007), education level (AOR = 0.720, *p* = 0.001), pregnancy status (AOR = 1.973, *p* < 0.001), and use of contraception (AOR of 0.489, *p* < 0.001). Our findings suggest that WRA in Tanzania aged above 35 should be the target population to accept the more tailored interventions.

## 1. Introduction

Anemia remains prevalent in more than one in three women of reproductive age (WRA) globally [1,2]. The burden is higher in low- and middle-income countries (LMICs) compared to the wealthier countries owing to socio-demographic disadvantages, among other factors. Even in high-burden countries, the brunt of anemia is not evenly distributed among women across the socio-demographic divides. Notable efforts have been put in place to mitigate anemia as an important risk factor for maternal and child health, with various levels of success. In the East African region, for example, the burden of anemia among WRA has declined significantly from 40% in 1995 to 28% in 2011 among non-pregnant women and from 46% in 1995 to 36% in 2011 among pregnant women [1]. The remaining burden is unprecedented because more than one-third of these women are at risk of health complications and subsequent challenges in child health and development [3,4,5,6].

Addressing the burden of anemia among WRA remains a cornerstone to attaining global maternal and child health targets. Such efforts can also help ameliorate the burden of child anemia [7], which is also at an unprecedented proportion [1]. To this end, addressing maternal anemia can also alleviate the current burden of maternal mortality [8]. Many countries, particularly those of low- and middle-income, could not attain the previously set global targets for maternal mortality [9,10].

In LMICs, including Tanzania, anemia among WRA arises primarily from iron deficiency [11]. Efforts to address anemia in Tanzania have not yielded substantial results, and anemia has, therefore, remained prevalent among women of reproductive age, pregnant women, and children in most regions of the country [12]. A recent survey indicated anemia is prevalent among 45% of WRA in Tanzania [13]. However, like other forms of undernutrition, the prevalence of anemia varies within and between the regions and other demographic divides. With such varying burdens, evidence on trends, characteristics, and determinants thereof are important in addressing such burdens and developing tailored interventions suitable for the country. We, therefore, analyzed the nationally representative datasets from 2005 to 2015 to address the mentioned scientific gaps.

## 2. Materials and Methods

### 2.1. Study Design

We analyzed secondary data from nationally representative samples of the Tanzania Demographic and Health Surveys (TDHS) conducted in 2004–2005 [14] and 2015–2016 [13]. Prior to this period, TDHS did not have variables for anemia, the primary outcome variable for this study.

The primary data collection of TDHS followed and adhered to all ethical considerations, including informed consent, and permission to use the data was sought through the DHS website (The DHS Program. Available online: https://dhsprogram.com (accessed on 1 May 2022)). The current analysis did not need additional IRB approval.

### 2.2. Data Sources

Data from the two surveys were obtained through cross-sectional studies conducted in all regions in Tanzania mainland and Zanzibar by the National Bureau of Statistics (NBS) and Zanzibar Bureau of Statistics (ZBS) under the United States Agency for International Development (USAID) funding and technical leadership of MEASURES. DHS surveys have been conducted every four years in Tanzania, as in some other LMICs, since 1992. The survey aims to examine and follow up on maternal and child health indicators. Variables in these surveys include household characteristics, feeding practices, reproductive and child indicators, HIV, malaria in children, and other related health indicators. Data are collected from women, men, and children in randomly selected households, making the sample nationally representative.

In these nationally representative surveys, the sample was selected in two stages. For the 2015–2016 survey, the first stage involved the selection of a total of 585 clusters (sample units, including Tanzania mainland and Zanzibar) consisting of enumeration areas (EAs). For Tanzania mainland, a total of 30 clusters were selected from Dar es Salaam, and 20 were selected from each of the other 24 regions. For the case of Zanzibar, a total of 15 sample points were selected from the 5 regions. The second stage of selection involved the systematic sampling of households. A listing for households was undertaken in all of the selected areas prior to the fieldwork. About 18 households were selected from each sample point for a total sample size of 10,496 households. Weighting factors were added to the data file so that the results would be proportional at the national level. For each survey, eligibility criteria included women and men ages 15–49 who were either permanent residents of the selected households or those who were defined as visitors through staying in the household the night before the survey.

### 2.3. Study Population and Sample Size

The current analysis included 23,203 WRA, defined as women aged 15–49 years. Of them, 10,139 WRA were from the 2004–2005 TDHS survey, while 13,064 WRA were from the 2015–2016 TDHS survey.

### 2.4. Measurement of Variables

The outcome variable was anemia among WRA defined by a low blood Hb level. Anemia was defined as having a serum Hb concentration below 11.0 g/dL in pregnant women and below 12.0 g/dL in non-pregnant women. Anemia among women was measured by collecting and testing capillary blood from a finger 122 prick with the HemoCue 201+ analyzer [13]. An adjustment of the Hb count was made for altitude with the following formulas: adjust = −0.032 ×alt + 0.022 ×alt2, adjHb = Hb-adjust if adjust > 0, where adjust is the amount of the adjustment, alt is the altitude in 1000 feet (converted from meters by dividing by 1000 and multiplying by 3.3), adjHb is the adjusted Hb level, and Hb is the measured Hb level in grams per deciliter. No adjustment was made for altitudes below 1000 m [13]. Cigarette smoking is associated with a generalized upward shift of the Hb distribution curve and has been found to reduce the utility of Hb levels to detect anemia [15]. Therefore, an adjustment was made for women who smoke (if the information was collected): for women who smoke less than 10 cigarettes per day, no adjustment was made; for women who smoke 10–19 cigarettes per day, adjust Hb (g/dL) concentration by −0.3; for women who smoke 20–39 cigarettes per day, adjust Hb (g/dL) concentration by −0.5; for women who smoke 40 or more cigarettes per day, adjust Hb (g/dL) concentration by −0.7; for women who smoke an unknown quantity or non-cigarettes smoking, adjust 132 Hb (g/dL) concentration by −0.3 [16]. The anemia variable was categorized as normal (Hb 11 or 12 g/dL and above for pregnant or non-pregnant women, respectively) or having anemia if otherwise. According to severity, anemia was categorized as having mild anemia (Hb 10–11.9 g/dL), moderate anemia (Hb 7–9.9 g/dL), and severe anemia (Hb < 7 g/dL). In pregnant women, mild anemia is defined as Hb count between 10.0 and 10.9 g/dL; moderate anemia is defined as Hb count between 7.0 and 9.9 g/dL; severe anemia is defined as Hb count less than 7.0 g/dL [1].

Independent variables included individual characteristics, household characteristics, and other health-related characteristics that are hypothesized to be related to anemia among WRA. The individual characteristics included age (in years), highest education level, marital status, and having any form of health insurance. The woman’s nutrition status was measured through Body Mass Index (BMI). BMI was calculated by dividing weight in kilograms by height in meters squared (kg/m^2^). A BMI < 16.5 was regarded as moderate to severe thinness; BMI between 16.5 and 18.5 as mild thinness; BMI between 18.5 and 24.9 as normal; BMI between 25 and 29.9 as overweight; and BMI of 30 and above as obese [13]. Household characteristics included the place of residence, whether urban or rural, number of household members, and weighted wealth index. Like in our previous studies, the weighted wealth index was computed using principal component analysis and factor analyses of the household assets ownership. The factor loadings, which are sample weights, are summed to generate the weighted wealth index. DHS data present the quintiles of such weighted wealth index categories as poorest, poorer, middle, richer, and richest.

Reproductive and child health characteristics were also included as independent variables. These were current pregnancy status, current breastfeeding, the number of children ever born from the respondent, contraception use status (13), and age of the respondent at the first childbirth.

### 2.5. Statistical Analyses

Analysis was conducted using both bivariate statistics and multiple regression analyses. For descriptive statistics, Pearson’s Chi-square test was used to examine the differences in the severity of anemia between the survey periods. GIS was used to map the changes in the regions between the two study periods. The resulted color-coded regional changes displayed the changes between the 2004/2005 and 2015/2016 surveys.

Logistic regression analysis was conducted to examine the effects of independent variables in the change of anemia among WRA in the two survey periods. In doing so, we adjusted for important confounders, including demographic, households, and health-related variables, to determine the adjusted decline. Furthermore, a two-level hierarchical logistic regression analysis was used to examine the remaining factors associated with anemia among WRA in Tanzania. This was achieved through multiple logistic regression analysis that involved the outcome variable as anemia among WRA and independent variables, including socio-demographic, household, and health-related characteristics, using the TDHS 2015–2016. Sampling weight was generated by the TDHS to handle the sampling design. Data were analyzed using Stata version 15 software (Texas, TX, USA).

## 3. Results

The analyzed evidence showed a modest decline in anemia among WRA in Tanzania over a period of one decade (Table 1). While an overall reduction was only 3.6% (from 48.4% to 44.8%), it was nevertheless statistically significant (*p* = 0.007). A significant contribution to this decline was the moderate form of anemia (*p* < 0.001).

**Figure 1:**
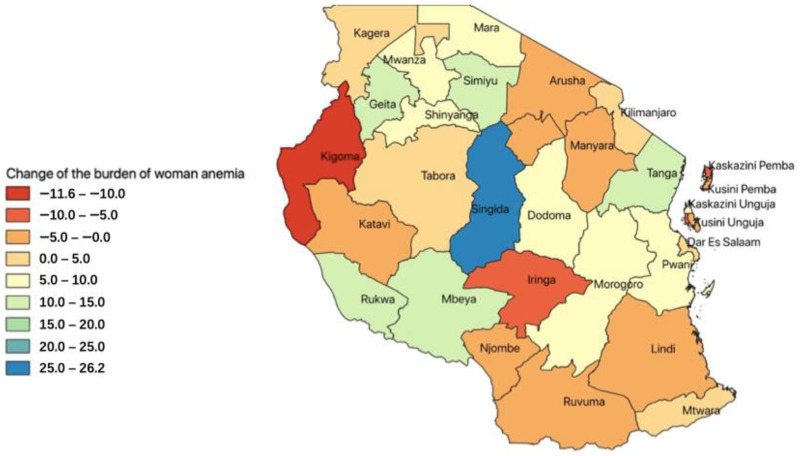
Geographic information system (GIS) mapping of the change in the magnitude of the burden of anemia in WRA (in percentage).

The decline of anemia varies across the regions in Tanzania. Singida region was noted to have a more significant decline in anemia (25%), while a total of 5 regions (Tanga, Rukwa, Mbeya, Simiyu, and Geita) observed a modest decline of between 10 and 20% (Figure 1). In the same period of time, two regions (Kigoma and Iringa) observed an increase in the burden of anemia.

The burden of anemia among WRA varies with age. However, such variation observed a u-shaped trend in both surveys (Table 2). While the burden declined with age in both data points, adolescents and older women have higher burdens compared to other age groups. Moreover, in both datasets, anemia maintains a declining trend with the increase in education level attained. For example, more than half of the uneducated women were anemic compared to 43% and 41% of women with secondary and higher education in 2005 and 2015, respectively. In both surveys and expected, women with wasting were more likely to succumb to anemia compared to those with normal BMI or overweight and obesity. Data on health insurance were not available in 2005. However, in 2015, WRA with health insurance had a lower prevalence of anemia (39%) compared to 45% among WRA with no health insurance. Anemia was also common in women who reside in households with more people, and also there was a growing trend of anemia with decreasing household wealth index in the 2015–2016 survey (Table 2).

As for maternal and child health characteristics, the burden of anemia is consistently higher among pregnant women in both data points. Compared to those who were not pregnant or not sure (43%) in 2015, about 57% of women who were pregnant during data collection were anemic. Compared to non-contraception users, women who used any form of contraception had a lower burden of anemia in both data points (Table 2).

After adjusting for confounders, the burden of anemia has declined among WRA over 10 years (Table 3). Tanzanian women were 14.5% less likely to suffer from any form of anemia in 2015 compared to 2005 (*p* = 0.005).

As for the remaining factors associated with anemia among WRA in Tanzania, after adjusting for confounders, there was no difference between urban and rural areas in anemia levels (Table 4). Compared to small-sized households (1–3 people), women living in households with 7–9 people were 1.5 times more likely to have anemia (*p* = 0.001). Moreover, those in households with 10 or more were almost twice as likely to succumb to anemia (*p* < 0.001). There was no significant association between anemia and wealth index. Compared to young women (aged below 20 years), women aged 35–39 and 40–44 were 1.5 times more likely to have anemia (*p* = 0.007 and *p* = 0.010, respectively). Education level was potentially a protective factor whereby, compared to uneducated women, those with primary and secondary education levels were 28% (*p* = 0.001) and 40% (<0.001), respectively, less likely to succumb to anemia. Women who were pregnant were more likely to have anemia [AOR 1.9, 95% CI (1.544–2.521), *p* < 0.001]. Compared to women who do not use any form of contraception, those who use modern contraceptive methods were 51% less likely to have anemia *p* < 0.001 (Table 4).

## 4. Discussion

The burden of anemia among WRA in Tanzania has declined in the past decade from 48.4% in 2005 to 44.8% in 2015. This decline is largely due to a decline in moderate anemia from 30% to 24.7% in the survey periods. Based on multiple logistic regression analyses, after adjusting for confounders, Tanzanian women were 14.5% less likely to suffer from anemia in 2015 compared to 2005. The burden of anemia among WRA in Tanzania is still high, and the trend is not matching the efforts and investments made in health across the country. Such investments made in Tanzania include improving health facilities infrastructures, the number of care workers, and making essential medicine available. The trend of a persistently high burden of anemia among WRA is similar in other low- and middle-income countries [1,17,18], costing many lives that could otherwise be prevented [19].

Like in other forms of undernutrition in Tanzania [20,21], the magnitude of anemia among WRA in Tanzania varies across regions. The decline has also assumed different rates over the same period. The Southern highland regions of Tanzania have high food productivity [22]. However, in this secondary analysis, some of these regions exhibited a modest to low decline in anemia (Rukwa, Mbeya, and Morogoro), while others (Iringa, Ruvuma, and Njombe) even observed an increase in anemia over the same period of time. This inconsistency might highlight the importance of dietary diversity, for high food productivity might still not guarantee the adequate intake of the micronutrients needed for the production of red blood cells and hemoglobin, which is a primary cause of anemia in Tanzania [23,24,25]. Most of the regions considered food basket regions of the country produce cereal-based foods and, to some extent, legumes. Most of the people in these regions, and, in particular, women, engage in farming activities, spending many hours on their farms, consuming mostly what they produce, and, therefore, jeopardizing adequate dietary diversity to prevent nutritional anemia [26,27,28]. The ideal minimum adequate diet would consist of both plant and animal sources of nutrients.

This study found a marked decline in anemia among WRA in the Singida region. Poor nutritional status in this region attracted a number of nutritional implementing partners. Through the coordination within the region, nutritional actions, including home gardens, food preparations, and other nutrition-sensitive interventions, led to a marked decline in just one decade [29].

As expected, anemia among WRA in Tanzania is associated with socio-demographic and household characteristics. For example, across the surveys, evidence suggests a unique u-shaped trend of anemia with the age of WRA. Like in other studies [30,31], the burden is higher among young women (15–24 years of age) and declines to the lowest level at 34 years of age, peaking in their forties. Adolescent females are at particular risk of anemia because of their iron needs for growth, as well as increased iron losses from menstruation. In Tanzania, the age at first childbirth is often below 19 years of age, which further exacerbates the risk of anemia as pregnancy incurs significant nutrient needs for iron to meet the requirements of expanding blood volume for the mother and, also, the growth and development of the fetus. To relieve the burden of anemia among adolescent females, efforts should be made to delay the age of first pregnancy among WRA [32,33]. Schools should provide access to comprehensive sexual and reproductive health (ASRH) education among girls, barriers to access to ASRH need to be addressed through adolescents’ friendly health services, communities should build up the norms of marriage until 18 years of age, and the government should enforce the laws to prohibit marriage of girls aged under 18 years. Women of older age were also fragile to anemia, which might be a consequence of frequent birth and nutritional challenges among the ageing populations owing to access to adequate iron supplies in the food they consume. Interventions should be made to avoid short birth-to-pregnancy intervals and address nutrition indicators for the ageing female population [34]. The health sector should provide health education on birth spacing and promote the use of contraception. Anemia is less prevalent among women who use contraception, according to this study.

The burden of anemia is also consistently higher among women with low or no education, women with lower BMI, women in low wealth quintile households, and women in large-sized families. Anemia in Tanzania, like other LMICs, is nutritional in nature [23,24,25]. Poverty, low levels of education, and large family sizes are more likely to cause to food shortages and, therefore, low dietary diversity. This can contribute to a low intake of iron-rich food sources and, therefore, the development of nutritional anemia. Findings of multiple logistic regression analyses show that, unlike other forms of undernutrition, anemia was not more prevalent in rural areas compared to urban areas. This is counter to findings from other contexts [35,36]; however, the burden among WRA in rural areas has declined significantly over 10 years compared to that of urban areas.

Evidence from this study should be carefully interpreted owing to the following three limitations. First, the evidence presented is from two cycles of DHS data and not panel data of similar participants. Therefore, decisive conclusions regarding trends in the same population cannot be firmly made without longitudinal analyses. Second, the 2015–2016 dataset had more variables that were not comparable with the 2005 dataset. Such variables included ownership of health insurance, post-natal clinic attendance, and deworming in the last pregnancy. Such variables are important, and, therefore, the inability to compare them poses a limitation to current reporting. Third, this was a secondary data analysis in which we could only analyze variables that were made available in the DHS design. This limits the conclusion made without adjusting all known covariates and confounders. Despite the limitations, this is the first study using big data in Tanzania to estimate the trends of anemia among WRA and examine the determinants thereof. Such findings are important not only for Tanzania but also for countries with similar contexts in the region and globally.

## 5. Conclusions

Tanzania has observed a significant but small decline in anemia (3.6%) among WRA over 10 years (2005 to 2015). This decline was not consistent across the regions, where regions with high food productivity have continued to succumb to a mild decline or increase in the burden of anemia. Efforts should be made to address this burden and increase the pace to ameliorate anemia, especially in regions with a poor decline. For all WRA, it is crucial to improve dietary diversification and increase the intake of foods that are rich in iron, vitamin A, vitamin B12, and folate. For women of younger age (15–24 years), daily iron supplementation is recommended to make up for the iron losses from menstruation. For pregnant women, iron and folic acid supplements early on in pregnancy are essential, accompanied by social and behavior-change communication strategies. Apart from the nutrition-specific interventions above, nutrition-sensitive interventions, such as improving the educational level among WRA, and enhancing reproductive health practices, including delaying the age of first pregnancy, ensuring access to quality maternal health care, and promoting optimal birth spacing through the use of contraception, remain important. Furthermore, empowering women with sustainable economics can have a long-lasting impact in the quest to address anemia and other health indicators among WRA in Tanzania and other LMICs. The findings in this study can provide a reference for international organizations for their future work on WRA anemia in LMICs.

## Figures and Tables

**Table 1:** The prevalence of anemia among women in Tanzania.

Anemia Status	2004–2005		2015–2016		Difference * (%)	*p*-Value
	N	% ^a^	N	% ^b^		
Normal	5236	51.6	7207	55.2		0.007
Anemic	4903	48.4	5857	44.8	−3.6	
Total population	10,139	100	13,064	100		
Mild	3309	67.5	4287	73.2	5.7	0.851
Moderate	1474	30.1	1446	24.7	−5.4	<0.001
Severe	120	2.4	124	2.1	−0.3	0.145

Note: Pearson’s Chi-square test was used to examine the differences in the severity of anemia between the survey periods. * Difference = % ^b^ − % ^a^.

**Table 2:** Individual, household, reproductive, and child health characteristics in relation to the changing burden of anemia among WRA in Tanzania.

Variable	Anemia in 2004–2005		Anemia in 2015–2016	
	No.	%	No.	%
Age (years)				
15–19	1082	49.0	1358	47.3
20–24	955	48.7	1131	46.3
25–29	898	48.6	900	42.8
30–34	675	44.5	704	40.8
35–39	513	49.4	739	46.0
40–44	412	50.1	602	45.0
45–49	369	49.3	422	43.2
Highest educational level				
No education	1352	54.9	979	51.2
Primary	3194	46.9	3609	44.5
Secondary	278	41.0	1204	41.8
Higher education level	78	42.9	65.0	41.3
Current marital status				
Never married	1062	45.8	1463	44.2
Married	3332	48.7	3628	44.9
Others	509	52.1	766	45.9
Woman’s nutrition status *				
Moderate to severe thinness	136	53.5	137	46.7
Mild thinness	350	50.0	413	49.3
Normal	3572	48.9	3850	47.2
Overweight	652	45.8	997	40.4
Obese	173	41.1	449	35.4
Has health insurance **				
No			5393	45.4
Yes			463	39.4
Type of residence				
Urban	1317	46.5	2083	44.5
Rural	3586	49.1	3774	45
Number of household members				
1–3	878	48.9	901	41.3
4–6	1832	44.7	2256	41.7
7–9	1281	48.8	1597	46.5
10+	913	56.3	1103	54.2
Wealth index				
Poorest	970	53.4	1079	48.5
Poorer	1005	52.2	1042	46.2
Middle	910	47.6	1060	45.9
Richer	851	42.9	1147	41.2
Richest	1167	46.7	1528	43.7
Currently pregnant				
No or unsure	4278	47.2	5218	43.7
Yes	625	58.2	639	57.1
Currently breastfeeding				
No	3464	48.7	4239	44.3
Yes	1439	47.6	1618	46.3
Number of children ever born				
0	1193	47.7	1529	46.1
1	749	50.6	989	46.6
2	700	48.3	812	43.9
3	556	47.2	633	42.3
4+	1705	48.3	1893	44.3
Contraception use by type				
No method	4031	51.2	4345	49.2
Folkloric method	38	39.4	26	46.1
Traditional method	171	42.9	270	42.8
Modern method	664	37.4	1216	34.3
Age at the first childbirth (years)				
0–19	2466	48.9	2719	45.2
20–29	1217	47.9	1543	43
30–49	28	47.1	64	46.8

*: Measured through Body Mass Index by dividing weight in kilograms by height in meters squared (kg/m2); A BMI < 16.5 = moderate to severe thinness; BMI between 16.5 and 18.5 = mild thinness; BMI between 18.5 and 24.9 = Normal; BMI between 25 and 29.9 = overweight; BMI 30 and above is obese; **: Data were available for only one dataset.

**Table 3:** Decline in women’s anemia in relation to other factors using TDHS 2004–2005 and TDHS 2015–2016.

Variable	AOR	95%CI	*p*-Value
Survey year			
2004–2005	1.000		
2015–2016	0.855	0.766–0.953	0.005

Note: Multiple logistic regressions were used. AOR was adjusted for individual, households, maternal, and child health characteristics.

**Table 4:** Remaining factors associated with anemia among WRA using the recent TDHS 2015–2016.

Variable	AOR	95%CI	*p*-Value
Household characteristics			
Type of residence			
Urban	1.000		
Rural	0.895	0.749–1.069	0.222
Number of household members			
1–3	1.000		
4–6	1.142	0.937–1.393	0.188
7–9	1.498	1.186–1.891	0.001
10+	1.941	1.458–2.585	<0.001
Weighted wealth index categories			
Poorest	1.000		
Poorer	1.017	0.828–1.250	0.870
Middle	1.119	0.910–1.376	0.287
Richer	0.921	0.741–1.144	0.455
Richest	1.086	0.832–1.418	0.544
Individual Characteristics			
Age (years)			
15–19	1.000		
20–24	1.188	0.943–1.495	0.143
25–29	1.147	0.875–1.503	0.321
30–34	1.133	0.833–1.540	0.427
35–39	1.564	1.128–2.169	0.007
40–44	1.571	1.114–2.215	0.010
45–49	1.374	0.920–2.050	0.120
Highest educational level			
No education	1.000		
Primary	0.720	0.597–0.867	0.001
Secondary	0.608	0.473–0.781	<0.001
Higher education level	0.606	0.285–1.289	0.193
Current marital status			
Never married	1.000		
Married	1.303	1.019–1.666	0.035
Other *	1.440	1.079–1.923	0.013
Currently pregnant			
No or unsure	1.000		
Yes	1.973 ***	1.544–2.521	<0.001
Number of children ever born			
0	1.000		
1	0.952	0.737–1.231	0.710
2	0.793	0.583–1.079	0.140
3	0.739	0.528–1.036	0.079
4+	0.632	0.448–0.892	0.009
Contraception use by type			
No method	1.000		
Folkloric method **	0.963	0.337–2.754	0.944
Traditional method ***	0.922	0.665–1.277	0.624
Modern method ****	0.489	0.417–0.575	<0.001
BMI			
Moderately and severely thin	1.000		
Mildly thin	1.236	0.777–1.966	0.372
Normal	1.048	0.696–1.578	0.821
Overweight	0.738	0.478–1.141	0.172
Obese	0.516	0.321–0.829	0.006
Covered by health insurance			
No			
Yes	0.886	0.702–1.119	0.309

Note: Multiple logistic regression was used. AORs were adjusted for individual, households, maternal, and child health characteristics. *: divorced/separated, cohabiting, living together; **: use of herbs (traditional medicine); ***: withdrawal, rhythm method; ****: female sterilization, IUCD, pill, injectables, male condoms, implants, emergency contraception; Note: Multiple logistic regression was used. Coefficients are ORs from logistic regressions including all variables presented in table.

## Data Availability

All datasets are available upon request from the DHS website.
